# Supplementary material for: Circular RNA hsa_circ_0051246 acts as a microRNA-375 sponge to promote the progression of gastric cancer stem cells via YAP1
Source: PeerJ. 2023 Nov 30;11:e16523. doi: 10.7717/peerj.16523 (PMC10950207; doi:10.7717/peerj.16523)

Figure 4F, G, H-Ki67, PCNA, Bax, Bcl-2, GAPDH

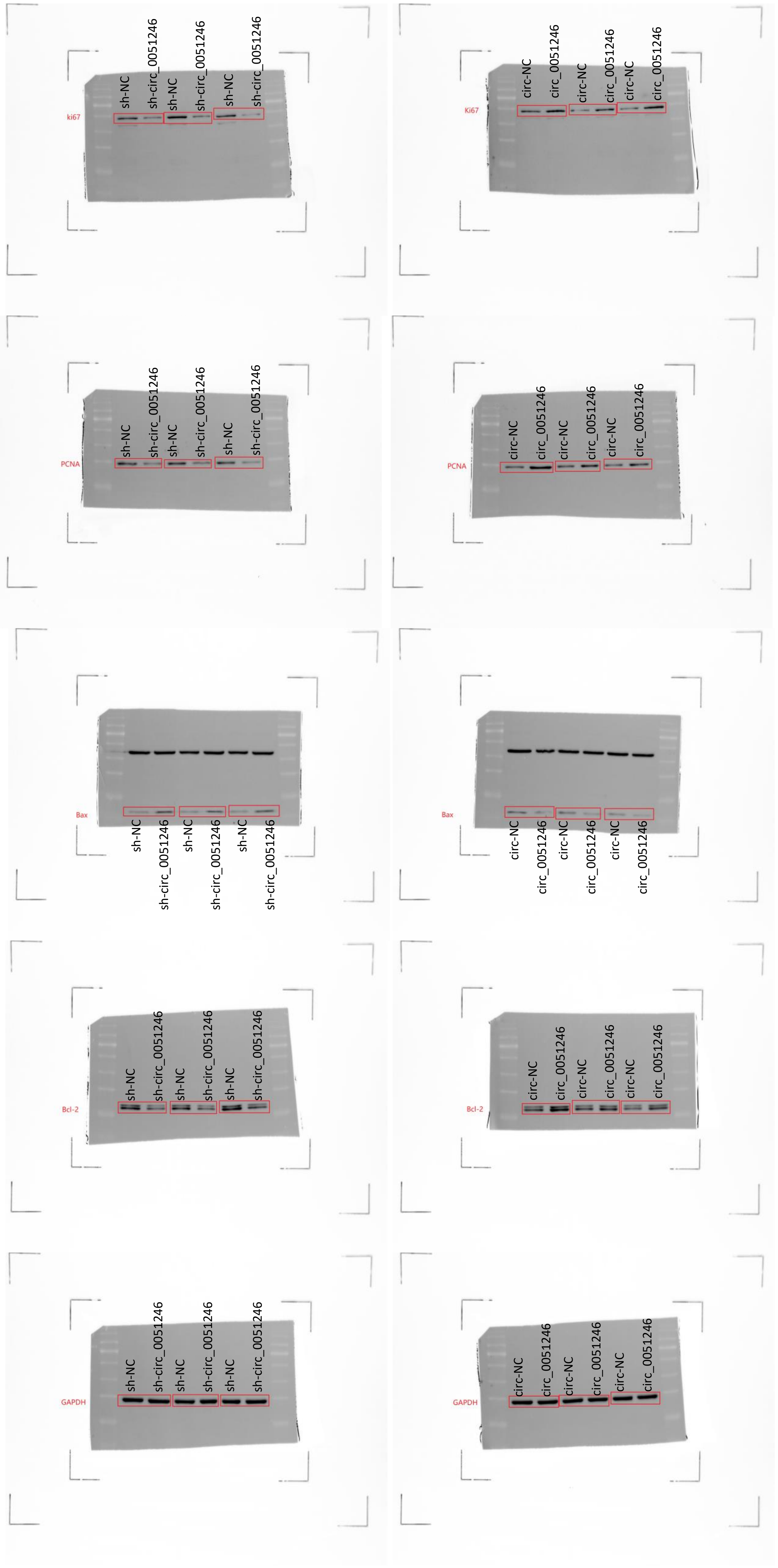

Figure 4F, G, H-N-cadherin, Vimentin, E-cadherin, GAPDH

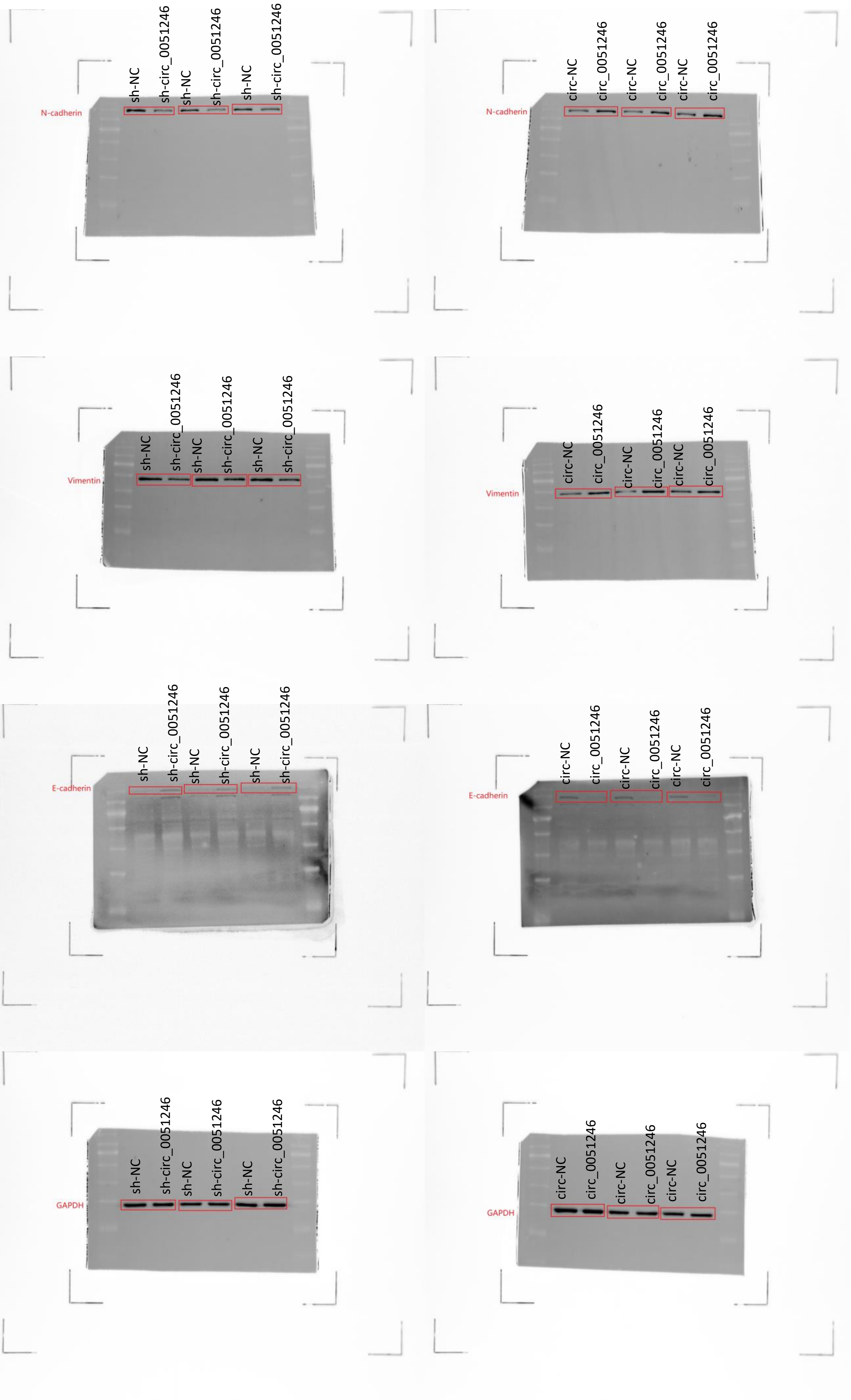

Figure 4F, G, H-YAP1, Notch 1, Jagged 1, GAPDH

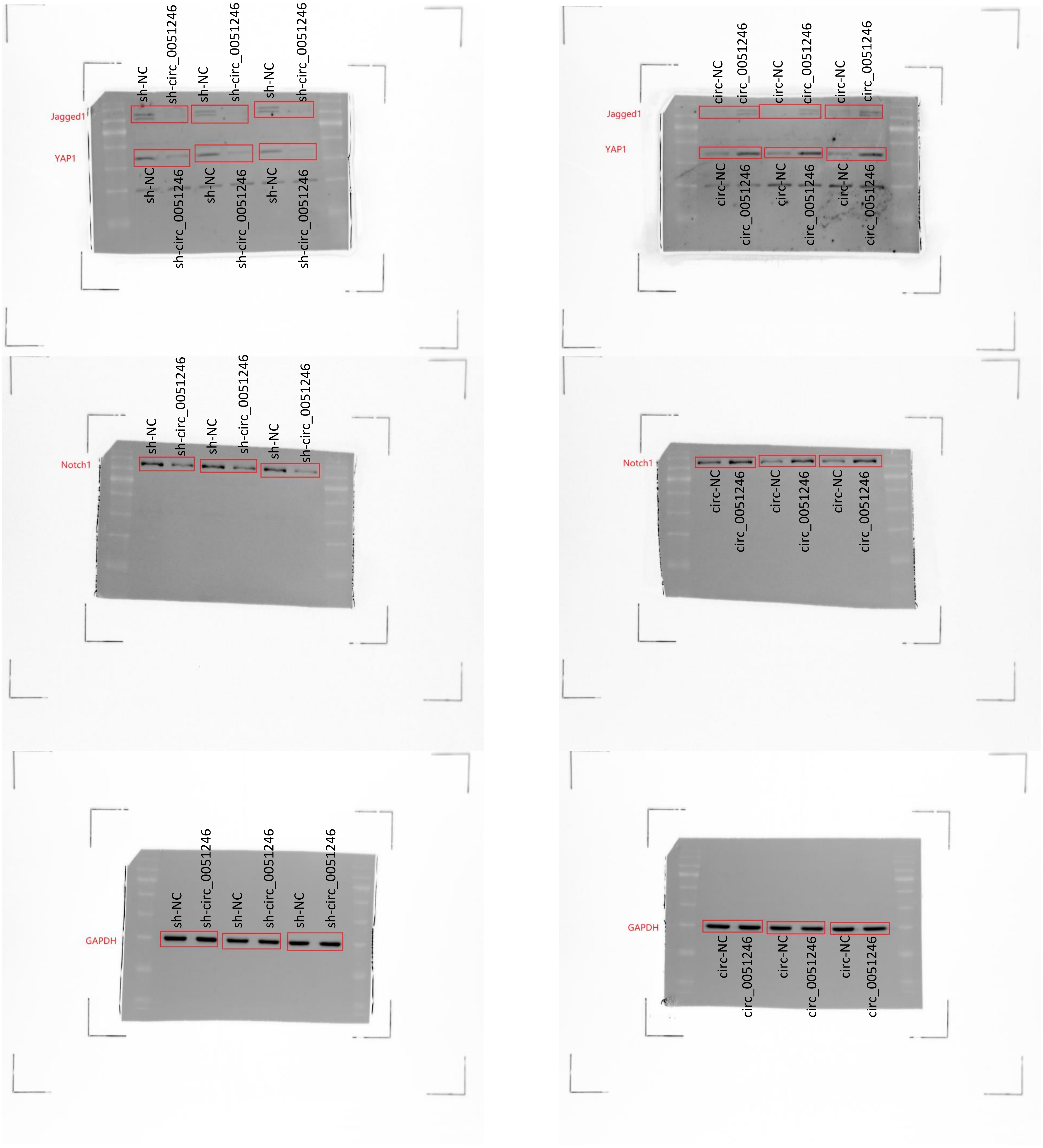

Figure 6C, D-Ki67, PCNA, Bax, Bcl-2, GAPDH;N-cadherin, Vimentin, E-cadherin, GAPDH; YAP1, Notch 1, Jagged 1, GAPDH

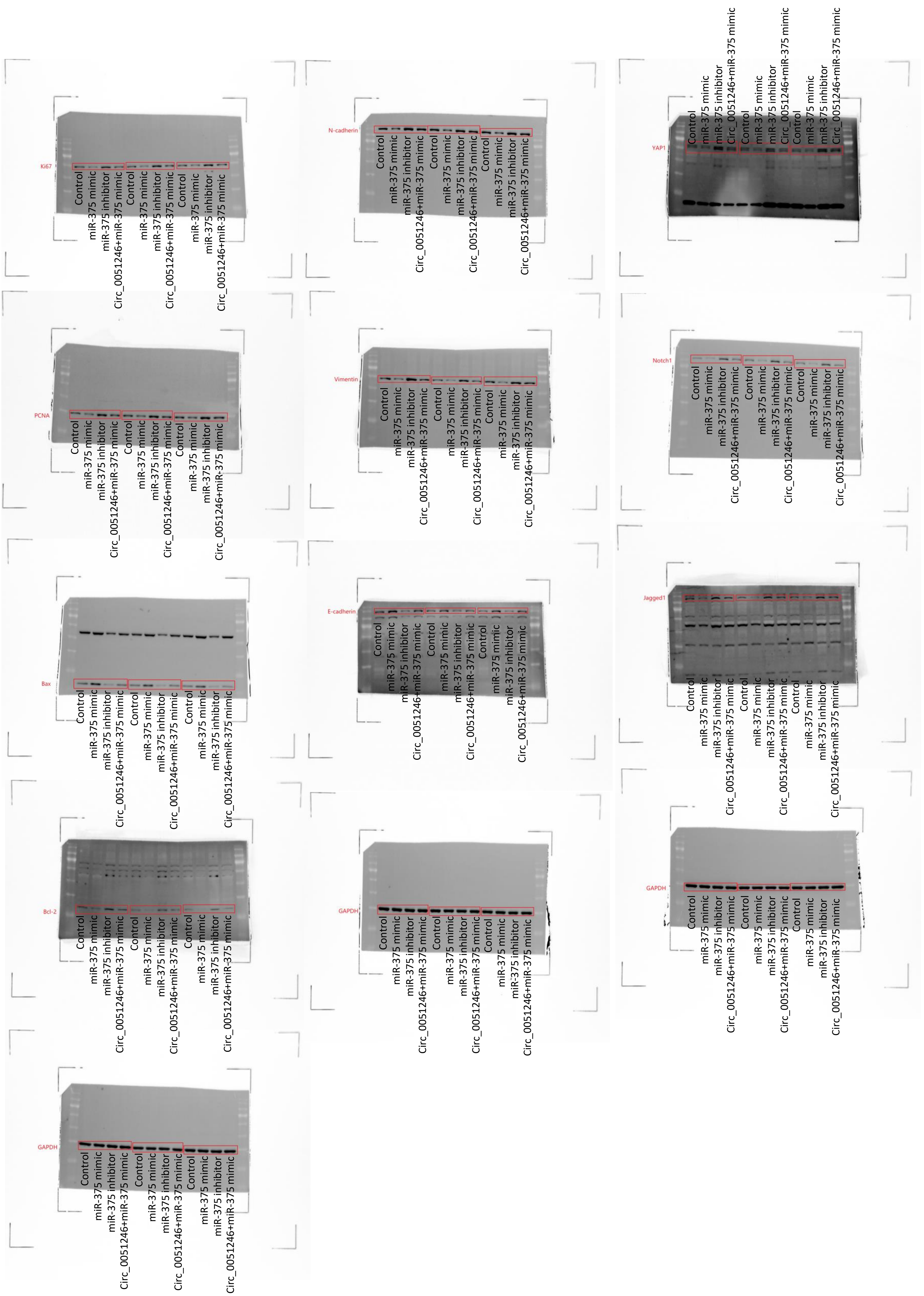

Figure 8C, D-N-cadherin, Vimentin, E-cadherin, GAPDH; YAP1, Notch 1, Jagged 1, GAPDH

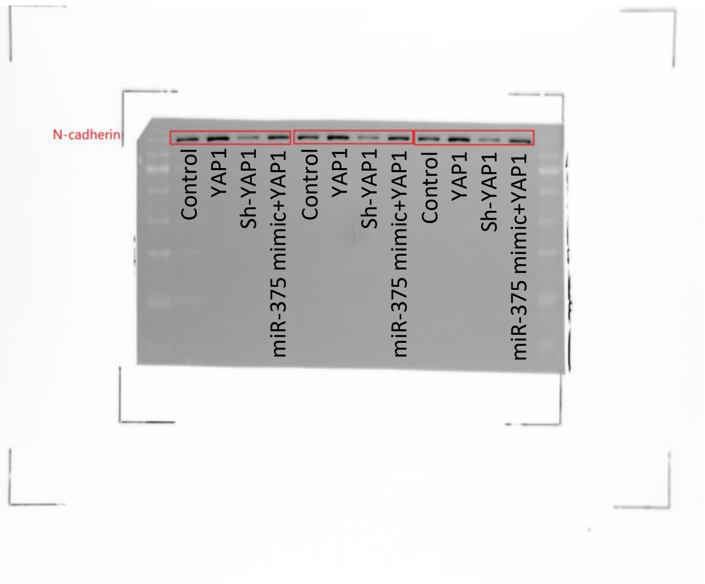

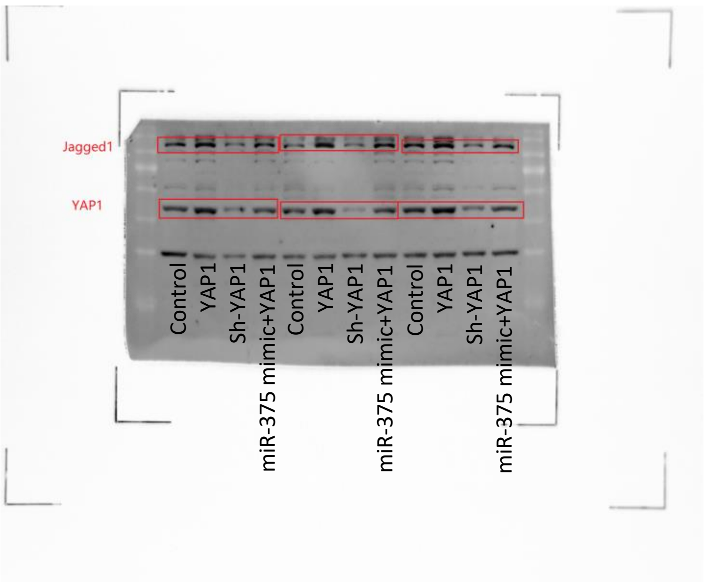

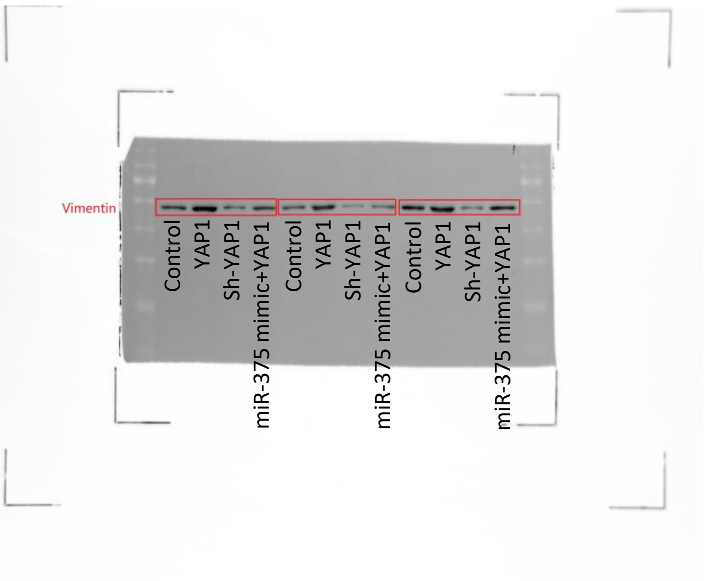

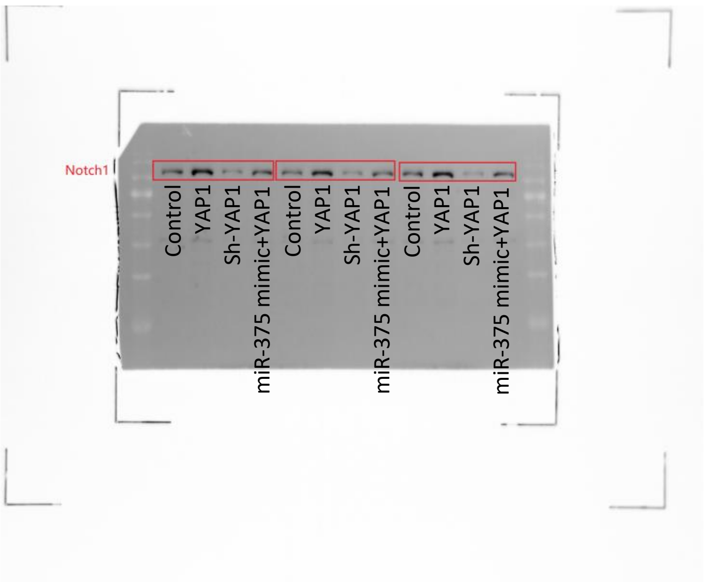

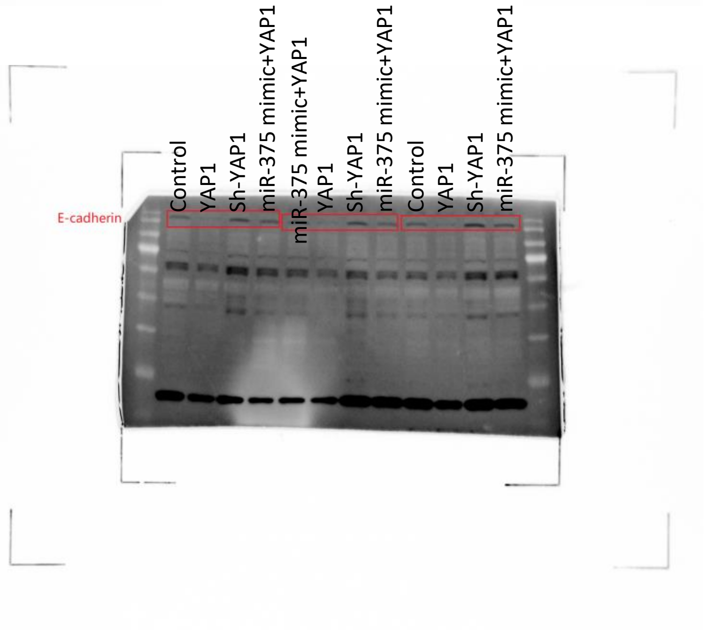

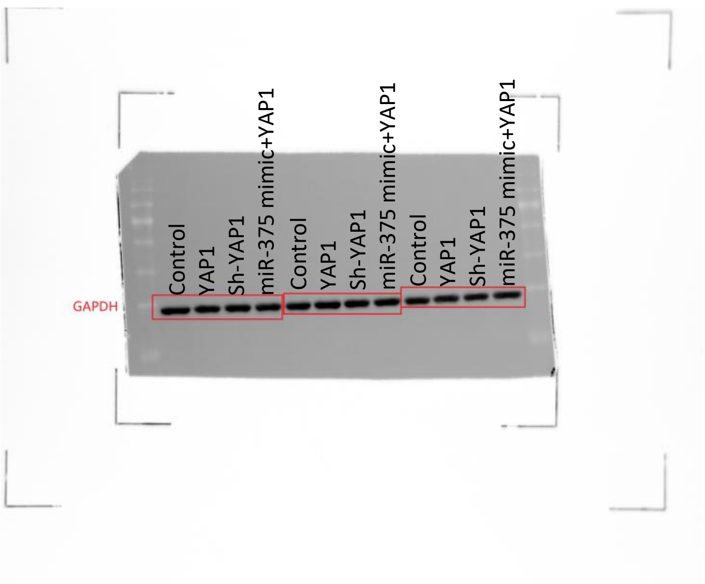

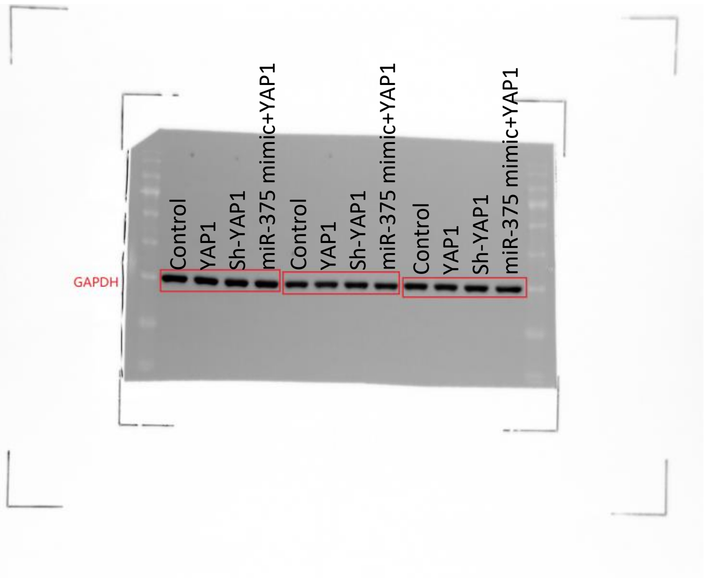

Figure 9F, G-YAP1, Notch 1, Jagged 1, GAPDH

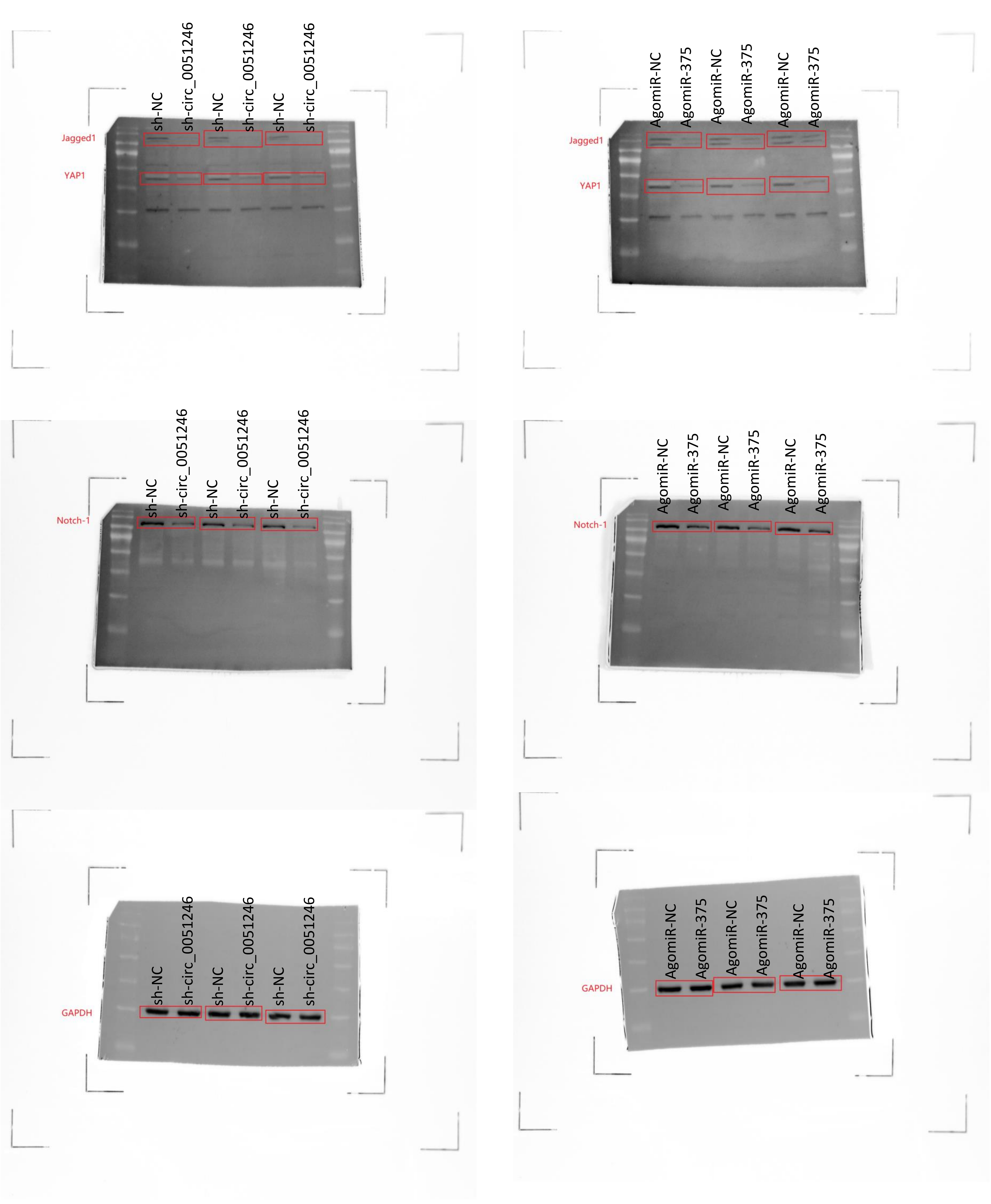

Supplement: File S1 [file peerj-11-16523-s002.pdf]
